# Amylin, Another Important Neuroendocrine Hormone for the Treatment of Diabesity

**DOI:** 10.3390/ijms25031517

**Published:** 2024-01-26

**Authors:** Stjepan Eržen, Gašper Tonin, Dubravka Jurišić Eržen, Jasna Klen

**Affiliations:** 1 Faculty of Medicine, University of Rijeka, 51000 Rijeka, Croatia; 2 Faculty of Medicine, University of Ljubljana, 1000 Ljubljana, Slovenia; 3 Faculty of Arts, University of Ljubljana, 1000 Ljubljana, Slovenia; 4 Department of Endocrinology and Diabetology, University Hospital Centre, 51000 Rijeka, Croatia; 5 Department of Internal Medicine, Faculty of Medicine, University of Rijeka, 51000 Rijeka, Croatia; 6 Division of Surgery, Department of Abdominal Surgery, University Medical Centre Ljubljana, 1000 Ljubljana, Slovenia; 7 Department of Internal Medicine, Faculty of Medicine, University of Ljubljana, 1000 Ljubljana, Slovenia

**Keywords:** diabetes mellitus type 1, diabetes mellitus type 2, obesity, diabesity, amylin receptor, peptide hormones, pramlintide, cagrilintide

## Abstract

Diabetes mellitus is a devastating chronic metabolic disease. Since the majority of type 2 diabetes mellitus patients are overweight or obese, a novel term—diabesity—has emerged. The gut–brain axis plays a critical function in maintaining glucose and energy homeostasis and involves a variety of peptides. Amylin is a neuroendocrine anorexigenic polypeptide hormone, which is co-secreted with insulin from β-cells of the pancreas in response to food consumption. Aside from its effect on glucose homeostasis, amylin inhibits homeostatic and hedonic feeding, induces satiety, and decreases body weight. In this narrative review, we summarized the current evidence and ongoing studies on the mechanism of action, clinical pharmacology, and applications of amylin and its analogs, pramlintide and cagrilintide, in the field of diabetology, endocrinology, and metabolism disorders, such as obesity.

## 1. Introduction

Diabetes currently affects more than half a billion people globally, with the number expected to more than double to 1.3 billion people in the next 30 years [1]. Type 2 diabetes mellitus (T2DM) is one of the most heterogeneous, devastating, and common chronic metabolic diseases [2,3]. In T2DM, several environmental and genetic factors contribute to impaired insulin production by pancreatic β-cells and impaired insulin sensitivity in tissues with consequent clinical manifestation of hyperglycemia [4]. On the other hand, the pathophysiology of type 1 diabetes mellitus (T1DM), which accounts for 10% of all cases of diabetes, classically involves the autoimmune destruction of β-cells in the pancreas by invading CD4+ and CD8+ T-cells and macrophages. Nevertheless, the pathogenesis of T1DM is very diverse since it is influenced by a spectrum of genetic, epigenetic, environmental, and immunological factors [5].

**Figure 1:**
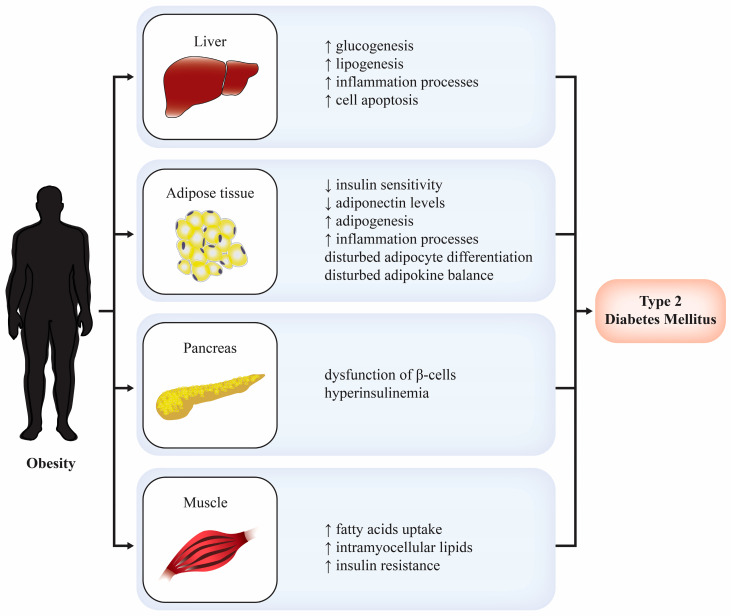
Pathophysiological connections between obesity and type 2 diabetes mellitus [2].

Obesity has been one of the most important global health issues in the last three decades, with approximately 800 million people currently affected. In addition, more than 90% of patients with T2DM in the age range of 40–59 are overweight or obese [3]. The pathogenesis of obesity is multifactorial, but a number of environmental risk factors are thought to critically contribute to the escalating obesity epidemic, notably unhealthy lifestyle behaviors such as excessive intake of processed foods and physical inactivity [6]. Due to the crucial role of obesity in the pathogenesis of T2DM, Sims et al. coined a novel term—diabesity—which describes the correlation between the two chronic metabolic diseases. Obesity and T2DM have various pathophysiological connections, as summarized in Figure 1. The primary mechanism in the development of diabesity is visceral adiposity, which leads to insulin resistance [7]. Various therapeutic approaches have been developed to address different pathophysiological mechanisms of this chronic metabolic disease.

Currently, the management of T2DM is personalized and based on glycemic targets with different clinical approaches such as glycated hemoglobin (HbA1c), self-monitored blood glucose (SMBG), continuous glucose monitoring (CGM), time-in-range (TIR), and glucose management indicator (GMI) [8]. Despite the numerous different drugs available for T2DM treatment, management of the disease is challenging for a variety of reasons. Some of them are drug side effects, contraindications to the use of selected drugs in kidney disease or heart failure, the risk of hypoglycemia, the inability to target every pathophysiological defect associated with hyperglycemia, and the common association of intensive glycemic control with the risk of weight gain. Therefore, any novel medicine for this prevalent chronic disease is appreciated.

Gut hormones control physiological processes in the pancreas, liver, adipose tissue, gut, and central nervous system, making them appealing therapeutic targets in the treatment of obesity and T2DM. The majority of the gut hormones are derived from enteroendocrine cells and have important roles in food digestion and absorption, insulin secretion, and appetite regulation. Bioactive peptides obtained from other types of intestinal epithelial cells have also been involved in metabolic control and can be termed gut hormones [9]. Glucagon-like peptide 1 (GLP-1) and gastric inhibitory polypeptide (GIP) are two major gut hormones that stimulate insulin secretion. Both are released after food ingestion and function to increase glucose-dependent insulin secretion [10]. Amylin or islet amyloid polypeptide (IAPP) is a centrally acting neuroendocrine anorexigenic hormone that is co-secreted with insulin by the pancreatic β-cells in response to nutrient ingestion [11].

In recent years, peptide-based agents that act on both glycemia and excess fat have been increasingly used in the treatment of T2DM and obesity. In this narrative review, we summarized the current evidence on the mechanism of action, clinical pharmacology, and applications of amylin in the field of diabesity. We have also outlined the current ongoing research in this field.

## 2. Amylin

### 2.1. Biochemical Structure

**Figure 2:**
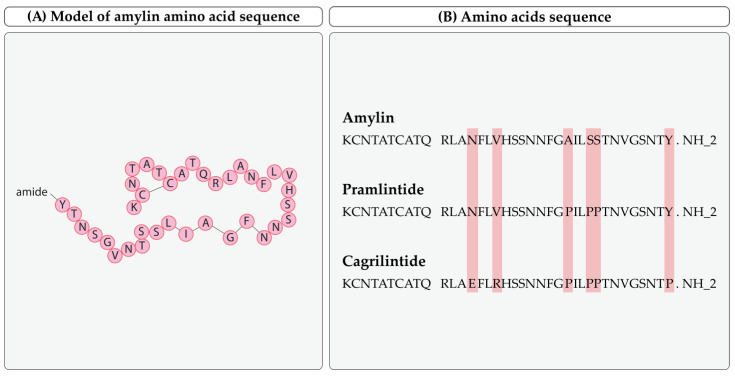
Chemical structure and amino acid sequence of amylin (amino acids are presented with their one letter abbreviations). (**A**) Model of amylin amino acid sequence. (**B**) Amylin, pramlintide, and cagrilintide amino acid sequences [14,15,16,17].

Amylin is a 37-amino-acid peptide (~4 kDa) with an intramolecular disulfide bridge between cysteine residues at positions 2 and 7 and an amidated c-terminus. It shares approximately 50% of the amino acid sequence with calcitonin gene-related peptides (CGRP α and CGRP β) and is structurally related to calcitonin. In blood, it can be found in non-glycosylated and glycosylated forms, with the latter inactive in blood [12,13]. The molecular formula of amylin is C54H95N19O19S [14]. Its amino acid sequence and comparison to analoges is presented in Figure 2.

### 2.2. Amylin Synthesis and Metabolism

Amylin is a part of the calcitonin gene family, which consists of calcitonin, CGRP, intermedin/adromedullin, and adrenomedullin (AM) [18]. It is already known that the amylin gene is located on the p arm of human chromosome 12. Primarily, amylin is synthesized as a preprohormone, which is then cleaved by prohormone protein convertase PC1/3 and PC2 into pro-amylin (proIAPP) [19]. To reach its full bioactivity, the prohormone undergoes amidation at the C-terminal and the formation of the disulfide between cysteine-2 and cysteine-7 [18]. Physiological plasma concentrations vary between 4 and 25 pmol/L, and it is primarily excreted via the kidneys.

Amylin metabolism appears to involve a dual process of renal clearance and proteolysis, forming different metabolites. Among them, a des-Lys metabolite is active, but other cleavage products are unlikely to provide active peptide fragments [20].

### 2.3. Physiology and Neuroendocrine Effects of Amylin

Amylin is important in numerous systems that regulate glycemic control, food intake, and body mass. Although the majority of current studies investigating the effects of amylin on various systems are based on animal models, it has been shown that amylin influences satiation, inhibits postprandial glucagon secretion, slows gastric emptying, and inhibits digestive enzyme secretion through various peripheral and central mechanisms. Besides its primary secretion from the pancreatic β-cells, a number of other tissues are also known to produce amylin, including the stomach, spinal ganglia, and the brain [21]. Synthesis and excretion of amylin from the pancreas are similar to insulin physiology as both peptides have a common regulatory promotor motif [22]. Therefore, it has been shown that the molar ratio of insulin to amylin secretion is somewhat constant, averaging approximately 2–5% [23]. The peptide is released after food ingestion and has a short half-time in plasma (~13 min) [24]. Its plasma concentration in the fasting state is 2–10 pM and 8–20 pM in the postprandial state [23].

Amylin suppresses postprandial glucagon secretion indirectly and directly through its effect on pancreatic α-cells. Accordingly, numerous studies show that it reduces hepatic glucose mobilization after nutrient ingestion [25,26]. Furthermore, it slows gastric emptying and pancreatic enzyme secretion, further lowering peak glucose levels in the circulation. Apart from its effects on peripheral tissues, it directly targets different structures in the central nervous system.

One of the most thoroughly researched functions of amylin is its effect on satiation, caused by the activation of noradrenergic neurons in the glucose-sensitive area postrema (AP) of the medulla oblongata [27]. Nerve fibers containing amylin have also been found in the nucleus tractus solitarius (NTS) and the lateral and parabrachial nucleus (LPBN), which are secondary sites of amylin action. These two sites send the neuronal signal to the lateral hypothalamus and its other parts, modulating the sensation of satiation [19,28]. In rats, acute peripheral administration of amylin leads to the quick reduction of bioenergetic status due to the reduced ingested meal size [29,30]. Direct brain infusions have the same effect, which is dose-dependent and blocked by amylin receptor antagonists [19].

Additionally, it has been shown that amylin could improve leptin sensitivity in obesity. Leptin is a hormone secreted by the adipose tissue, signalizing the level of triglycerides in the adipose tissue. In rats, simultaneous exogenous administration of leptin and amylin leads to weight loss, although, in more severe obesity, their synergistic effect was less prominent [27,31]. Moreover, amylin administration in animal models has been shown to increase energy expenditure [32,33].

This polypeptide hormone is also thought to affect the cardiovascular system as it causes vasodilatation, which decreases blood pressure and activates baroreflex, raising the heart rate in rodents. Its vascular effects in animal models are believed to be mediated by the CGRP receptors, which are extensively expressed on the endothelium. However, as amylin potency on this receptor is relatively small, very high plasma concentrations of amylin are needed to cause an effect [24].

Although the physiological effects of amylin in humans are poorly researched, the clinical trials with amylin analogs might shed light on its role in the human body.

### 2.4. Role of Amylin in the Pathogenesis of Diseases

Apart from its role in the energy and glucose homeostasis in the body, amylin is important in the pathogenesis of various diseases due to its ability to self-aggregate. It is soluble in its unfolded, monomeric state. However, intracellularly, it can form oligomers that can aggregate. Namely, oligomers show misfolded α-helix conformation that can, under certain conditions, further transform to β-sheet-rich fibrils, forming amyloid deposits that might have toxic effects in the various tissues where amylin is expressed [34].

Although its aggregates might have a role in the pathogenesis of some of the neurodegenerative diseases (for example, Alzheimer’s disease and Parkinson’s disease), we have focused on the role they play in diabetes and obesity [35,36,37].

#### Amylin’s Role in Diabetes and Obesity

In T1DM, amylin basal levels are reduced, and the postprandial secretion is diminished [38]. However, in T2DM, increased insulin resistance is present in the peripheral tissues. Consequently, there is more insulin secreted from the β-cells in the pancreas. Along with insulin, amylin production is increased, with significantly higher levels of amylin observed in the fasting (40 pM) and postprandial states (90 pM). Nonetheless, as β-cells become exhausted in the later stages of the disease, amylin secretion diminishes [23,25]. Meanwhile, increased secretion of amylin may lead to abnormalities in the processing of amylin, resulting in amyloid deposition in the β-cells. At first, amyloid is present intracellularly but is deposited extracellularly with disease progression and β-cells death. It has been shown that amyloid can be found in the pancreas of more than 90% of T2DM patients. Several hypotheses have been postulated to explain how these amylin aggregates can cause β-cell loss due to their toxic effects [27]. Notable proposed mechanisms include endoplasmic reticulum correction mechanisms overloading, disturbance of the intracellular cell transport, disruption of the organelles membranes (e.g., mitochondria), and inflammasome activation [27].

Amylin affects satiation, energy expenditure, and body weight. In adiposity, higher plasma concentrations of amylin were observed, which may imply that amylin signals the level of adiposity [39,40]. Even in obesity, food intake leads to an increase in the amylin concentration in plasma. Moreover, it seems that amylin sensitivity is not reduced in this state since exogenous administration of amylin leads to reduced appetite within a few minutes. Although the role of amylin in obesity has been modestly researched in animal models, more studies are needed to clarify its role in human diseases [24].

### 2.5. Amylin Receptor

Amylin binds with high affinity to more than one distinct receptor. Moreover, the structure of various amylin receptors is somewhat complicated. Additionally, it is essential to note that amylin receptors are similar to calcitonin receptors when interpreting the pharmacokinetic and pharmacodynamic properties of the hormone [24].

Studies analyzing the binding sites of amylin have shown an overlap with calcitonin binding sites, raising questions about the connections between the two [24,41]. On the other hand, not all the cells that expressed calcitonin receptors (CTR) have interacted with amylin. This provoked the idea that other factors apart from CTR are behind the response difference to the amylin molecule. Accordingly, researchers have shown that other peptides, namely receptor activity-modifying protein (RAMP), might be responsible for this effect [42,43]. Although the calcitonin receptor does have an affinity for amylin and can be activated by it, the RAMP peptide promotes the binding and increases the potency of amylin receptors, thus enhancing its action [44].

Apart from the CTR that also responds to amylin, several other amylin receptors are more structurally intricate. They exist as a CTR/RAMP complex containing CTR, a G-protein-coupled receptor (GPCR), and receptor activity-modifying protein (RAMP).

Calcitonin receptor (CTR) is a core family B GPCR with seven transmembrane segments. It has two major splice variants, hCT(a) and hCT(b), with the second one being more prevalent [45]. The variants differ in the deletion of 48 base pairs that encode 16 amino acids in intracellular domain 1 [46]. RAMP is a peptide important for transporting CTR to the cell membrane and regulating its ligand specificity [47,48,49]. There are three types of RAMP (RAMP1, RAMP2, and RAMP3), which share 31% of their amino acid sequence and have a similar basic structure [24]. They consist of a large extracellular N-terminal domain with a signal peptide and four cysteines, a transmembrane domain, and a shorter C-terminal domain with approximately 10 amino acids. RAMP1 and RAMP3 are 26 amino acids shorter than RAMP2 [50].

**Figure 3:**
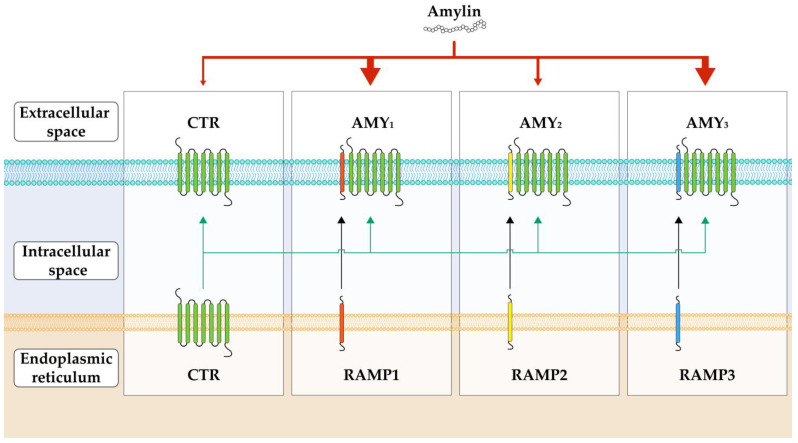
Schematic representation of the complex relationship between calcitonin receptor (CTR), receptor activity-modifying proteins (RAMP), and three main amylin receptors (AMY_1_, AMY_2,_ and AMY_3_). Two main splice variants of CTR (in the figure, we have presented only one for simplicity) and different RAMPs (RAMP1, RAMP2, and RAMP3) form the CTR/RAMP complex, also known as the AMY receptor. In this complex, RAMP modifies and enhances the susceptibility of the CTR for amylin. As there are different RAMPs, there are multiple variants of AMY receptors, namely AMY_1_, AMY_2_, and AMY_3._ Amylin has the strongest potency for the AMY_1_ and AMY_2_ and variable potency for the AMY_3_ and the non-complex form CTR (as shown by the red arrows at the top of the picture).

The switch of CTR to the amylin receptor phenotype depends on the receptor isoform and cellular background [42,43,44,50]. Since there are two major variants of CTR and three major RAMPs associated with amylin receptors, there are six amylin receptor subtypes (AMY_1a_, AMY_1b_, AMY_2a_, AMY_2b_, AMY_3a_, AMY_3b_) with different pharmacological properties and physiological relevance. Nevertheless, the three most important variants of amylin receptors are determined by the type of RAMP molecule: AMY_1_, AMY_2_, and AMY_3_. This complex relationship between the CTR and RAMP at the cellular level is illustrated in Figure 3. Amylin has a high potency for AMY_1_ and AMY_3_ and variably for AMY_2_ [51].

Although the CTR/RAMP complex signal pathway has not been widely researched, available evidence indicates that AMY receptors cause the generation of cAMP and cGMP and possibly the mobilization of intracellular calcium. Since CTR receptors also couple with multiple G proteins (e.g., Gαs, Gαq, Gαi/o), the AMY receptors could also modulate the activation of various kinases (including ERK1/2 and FAK) [19,52]. Therefore, different configurations of CTR and RAMP within the complex might dictate the activation of one or more of the various intracellular pathways, leading to the expression of the physiological effects. Some of the receptor antagonists are truncated forms of an endogenous protein or its variants. The most known amylin receptor antagonists are salmon calcitonin 8–32 and AC187 [24].

As mentioned, the binding sites of amylin are similar to calcitonin binding sites due to the discussed structural resemblance of their receptors. However, determining the expression of amylin receptor subtypes and their physiologic relevance in humans is a great challenge due to numerous factors, such as the structural heterogeneity of the receptor. Nevertheless, in mainly animal models, it has been shown that it is expressed in the various areas of the CNS and in different major cell types (neurons, glial cells, endothelial cells). Some of these areas include the subfornical organ, nucleus of the solitarius tract, nucleus accumbens, hypothalamus, and ventral tegmental area. In humans, the receptor was shown to be expressed in blood vessels and spinal trigeminal tract [53,54,55,56].

## 3. Amyline Analogs

### 3.1. Pramlintide

The so-called amylinomimetic agent, pramlintide, is a synthetic amylin analog developed around 1995, which binds to the amylin receptors with equipotent agonistic effects as human amylin. The drug is available for both T1DM and T2DM. It is solubilized at an acidic pH of 4 and administered subcutaneously during mealtime. Postprandial glucovariability results not only from insulin deficiency but also from glucagon surplus. Pramlintide modulates the level of postprandial glucose by suppressing the postprandial rise of glucagon, inhibiting gastric emptying, and promoting satiety and reduction of food intake via a central mechanism. This drug is used with insulin when postprandial goal levels of glucose cannot be reached [57].

#### 3.1.1. Pharmacokinetics

Subcutaneous absorption of pramlintide is most reliable when administered in the abdomen and thigh compared with the arm. Pramlintide reaches peak serum levels within 30 min of administration and has linear pharmacokinetics. Its bioavailability ranges from 25% to 40%. The drug is mostly eliminated via the kidneys, with an elimination half-life of 30–50 min. Its concentrations have been shown to be higher in those with kidney failure; nevertheless, it is approved for use in patients with mild to moderate kidney dysfunction with eGFR > 20 mL/min/m^2^ [58].

#### 3.1.2. Administration and Dosage

Pramlintide must be injected just before mealtime: 15 to 60 μg subcutaneously in patients with T1DM and 60 to 120 μg subcutaneously in patients with T2DM [59]. The dosage of 15 µg is predicted to be equivalent to 2.5 units of insulin [60].

It is commercially available in combination with insulin. Due to its short half-life, it is used in three subcutaneous injections per day. In contrast to insulin, pramlintide eliminates the need for mealtime dose adjustments. Its broad therapeutic range enables patients to maintain a consistent dose, irrespective of meal size, carbohydrate content, or blood glucose concentrations. However, further studies are needed to assess the efficiency and safety of pramlintide and insulin combination in T1DM and T2DM [59].

#### 3.1.3. Clinical Use

Although pramlintide lacks a clear recommendation in the guidelines, it may be considered an alternative or adjunct to rapid-acting insulin analogs. It might be used in patients with insufficient glycemic control who have already received basal insulin therapy and require additional short-acting agents but aim to avoid weight gain [61]. Pramlintide primarily impacts postprandial hyperglycemia excursions, with little effect on mean glycemic control as determined by HbA1c [57]. A summary of clinical studies of pramlintide treatment in patients with diabesity is presented in Table 1.

#### 3.1.4. Adverse Effects

The main adverse effects of pramlintide are generally mild to moderate gastrointestinal symptoms, such as nausea, vomiting, and anorexia, which disappear four weeks after drug use. A low initial dose decreases the possibility of nausea, but in a small number of patients, this adverse effect occurs even in lower doses. This suggests that nausea is a consequence of a central effect [57]. However, one study reported other adverse effects, such as headache, inflicted injury, and sinusitis. Furthermore, none of the major studies showed liver, kidney, or cardiac toxicity, but a potential impact on bone mineral density has been suggested [62]. Although hypoglycemia is not a side effect of pramlintide, caution is needed when used with therapies with known hypoglycemic effects, such as insulin. Due to the risk of hypoglycemia, the initial treatment doses of insulin administered simultaneously with pramlintide must be lowered by 50% or more [59]. Nevertheless, in studies with T2DM patients, hypoglycemia was less common [62,63].

#### 3.1.5. Contraindications and Carcinogenicity

Pramlintide is contraindicated in patients with gastrointestinal diseases and those undergoing therapy with alpha-glucosidase inhibitors due to the increased risk of nausea [57]. Similarly, the drug should not be administered to patients with confirmed gastroparesis. Special caution is also required in those T1DM patients who are unaware of their hypoglycemias.

Pramlintide may interact with different drugs, such as oral anti-diabetic drugs, insulin, α-glucosidase inhibitors, anticholinergics, angiotensin-converting enzyme inhibitors, fibrates, salicylates, disopyramide, fluoxetine, monoamine oxidase inhibitors, pentoxifylline, somatostatin analogs, and sulfonamide antibiotics [64,65].

**Table 1:** Summary clinical studies in patients with diabesity treated with pramlintide. T1DM—type 1 diabetes mellitus; T2DM—type 2 diabetes mellitus; HbA1C—hemoglobin A1C.

Authors	Research Design (Primary Goal, Duration)	Number of Patients	Results	Main Conclusions
Karl D et al., 2007 [66]	Open-label study. Safety and efficacy of pramlintide therapy. Duration: 2 years.	166 insulin-treated patients with **T2DM**	The change in HBA1c from baseline was −0.56%. Pramlintide therapy notably reduced weight (−2.8 kg) and postprandial glucose excursions.	Pramlintide initiation and mealtime insulin reduction led to weight loss. It also improved postprandial glucose excursions and HBA1c.
Pencek R et al., 2010 [67]	Open-label, multicenter, observational study. Primary goal was to assess the risk of insulin-induced severe hypoglycemia after pramlintide initiation. Duration: 6 months	1297 patients with **T1DM** and **T2DM** with inadequate glycaemic control	After 3 months, the incidence of patient-ascertained severe hypoglycemia (PASH) was 2.8% in patients with T2DM and 4.8% in patients with T1DM.	The possibility of insulin-induced severe hypoglycemia after pramlintide therapy initiation is low in patients with T2DM or T1DM.
Riddle M et al., 2007 [68]	A randomized, double-blind, placebo-controlled, multicenter study. Safety of adding pramlinitide to insulin glargine therapy. Duration: one year.	212 patients with **T2DM** using insulin glargine in addition to pramlintide or placebo	Reductions in HBA1c (−0.70% against −0.36%) and postprandial glucose increase were more significant in pramlintide-treated patients. Pramlinitide-treated patients experienced weight loss (−1.6 kg), while placebo gained weight (+0.7 kg).	Pramlintide improved HBA1c and postprandial glucose with weight reduction in T2DM patients.
Peyrot M et al., 2010 [69]	A randomized, open-label, parallel-group, multicenter study. The effectiveness of basal insulin regimens with rapid-acting insulin or pramlintide. Duration: 9 months.	112 patients with **T2DM** and basal insulin therapy in addition to pramlintide or rapid-acting insulin	Total diabetes distress in pramlintide patients improved significantly. On the other hand, patients with rapid-acting insulin did not. The perception of hypoglycemia was improved only in pramlintide patients.	Adding pramlintide to basal insulin treatment improved life quality and satisfaction compared with rapid-acting insulin analogs.
Whitehouse et al., 2002 [70]	A double-blinded clinical trial with parallel assignment. Effects of pramlintide on HBA1c and weight. Duration: 52 weeks.	480 patients with **T1DM**	HBA1c was lower in patients with pramlintide (−0.39%) in comparison with placebo (−0.12%). The patients with pramlinitide had a weight loss (−0.5%) in contrast to placebo patients with weight gain (+1.0%).	Pramlintide has a positive effect on HBA1c and weight loss compared to placebo.
Ratner et al., 2000 [62]	The study was double-blinded with parallel assignment. Effects of pramlinitide on weight and HBA1c. Duration: 52 weeks.	538 patients with insulin-treated **T2DM**	The patients with pramlintide had a weight loss (−0.3% to −1.3% depending on the dosage); on the contrary, placebo patients had a weight gain (+1.0%). HBA1c was lower in patients with pramlintide (−0.3% to −6.0% depending on the dosage) in comparison with placebo (−0.2%).	Pramlintide has a positive effect on weight loss and HBA1c compared with placebo.
Hollander et al., 2003 [63]	The study was double-blinded with parallel assignment. Pramlintide effects on HBA1c and weight. Duration: 52 weeks.	498 patients with **T2DM**	HBA1c was lower in patients with pramlinitide (−0.35% to −0.62% depending on the dosage) than a placebo (−0.22%). The patients with pramlintide had a weight loss (−0.5% to −1.2% depending on the dosage); on the contrary, placebo patients had a weight gain (+0.7%).	Pramlintide has a positive effect on HBA1c and weight loss compared to placebo.
Gottlieb et al., 1999 [71]	The study was double-blinded with parallel assignment. Effects of pramlintide on HBA1c and weight. Duration: 26 weeks.	499 patients with **T2DM**	The patients with pramlintide had a weight loss (−0.8% to −1.4% depending on the dosage); on the contrary, placebo patients had a weight gain (+0.1%). HBA1c was lower in patients with pramlinitide (−0.3% to −0.4% depending on the dosage) in comparison with placebo (−0.1%).	Pramlintide has a positive effect on HBA1c and weight loss compared to the placebo.

In animal models, pramlintide exposure did not result in tumor growth in any organ. In contrast, human studies have found that pramlintide increases the cytotoxic effects of conventional chemotherapy when targeting colorectal cancer cell lines [72] or thymic lymphoma tumors [73] containing wild-type or mutant p53. Additionally, one study showed an identical effect in osteosarcoma cells with high- or mid-range glycolytic activity. Tumor cell apoptosis might be a result of limited or decreased availability of glucose in the blood, which is required for rapid cancer cell growth [74].

#### 3.1.6. Cardiovascular Safety

Pramlintide, in addition to insulin, has an effect on weight loss, which is moderate in overweight patients and moderately obese patients and occurs regardless of the reduction of HbA1c. Weight loss is more noticeable in severe obesity patients and occurs even without nausea, which suggests that weight loss is not caused by nausea. Weight loss and reduction of appetite may indicate that pramlintide has a beneficial effect on inflammatory markers and dyslipidemia (significant reduction of total cholesterol and LDL levels). Nevertheless, it has no effect on blood pressure [57]. Similarly, the use of pramlintide in T2DM patients with inadequate glycemic control was not linked to an elevated cardiovascular risk. Interestingly, the pramlintide treatment group demonstrated a lower incidence of fatal cardiovascular events in contrast to the comparator group [75]. The incidence of major adverse cardiovascular events (MACE) noted in pramlintide studies (namely 4% over one year) closely resembled the MACE frequency observed in the ORIGIN cardiovascular outcome study with insulin glargine (approximately 3% per year) [76].

#### 3.1.7. Pramlintide and T1DM

Pramlintide treatment in T1DM might benefit highly motivated patients sub-optimally treated with basal-bolus insulin therapy or patients on insulin pump therapy with weight gain despite all recommended lifestyle changes [70,77].

When taken before meals and combined with preprandial insulin, it lowers postprandial hyperglycemia by decreasing postprandial hyperglucagonemia and delaying gastric emptying [57]. Moreover, when used with insulin, pre-mealtime administration of pramlintide significantly reduced HbA1c levels by 0.3–0.7%. Moreover, it decreased body weight by 0.4–1.4 kg [70,78,79].

Whitehouse et al. showed that pramlintide significantly reduces HbA1c compared to placebo at week 13 and week 52. Furthermore, they demonstrated greater weight loss in the pramlintide group [70]. A double-blind, randomized study demonstrated that pramlintide significantly reduced weight despite achieving a comparable reduction in HbA1c compared to the placebo [77]. Anderson et al. found that the co-formulation ADO09 of pramlintide and the insulin analog A21G reduced postprandial glucose excursions in the first hour post-meal by >95% compared to insulin lispro [80].

#### 3.1.8. Pramlintide and T2DM

Pramlintide is recommended in patients who are insufficiently managed with preprandial insulin and cannot control their weight with lifestyle changes [57]. A randomized clinical trial by Ratner et al. demonstrated that incorporating pramlintide (150 μg) into mealtime insulin led to a mean reduction of HbA1c by 1% in 13 weeks [62]. When used with insulin, pre-mealtime administration of pramlintide significantly reduces HbA1c levels by 0.3–1.0%. Moreover, it decreases body weight by 0.5–1.8 kg [62,63].

### 3.2. Cagrilintide

Since pramlintide is a short-acting amylin agonist, considerable effort has been invested in developing long-acting amylin agonists. The therapeutic role of cagrilintide is not fully understood due to limited research, and ongoing studies aim to elucidate its potential. Nevertheless, we have tried to summarize the available data about this promising drug.

Cagrilintide is a stable lipidated non-selective long-acting amylin analog administered subcutaneously weekly and is currently a potential agent in the treatment of obesity. It is a dual amylin and calcitonin receptor agonist (DACRA) based on salmon calcitonin and has demonstrated greater efficiency in obesity treatment, fasting glucose control, and HbA1c management in rat models compared to amylin therapy. This efficacy may be attributed to the significant role of the calcitonin receptor in glucose metabolism. Compared to another DACRA, KBP-366, both molecules activate calcitonin receptors in vivo and in vitro. However, KBP-336 exhibits greater potency than cagrilintide, demonstrating superior effectiveness in postprandial glucose control and weight loss [81].

There are several differences in the peptide structure of cagrilintide compared to pramlintide. The proline substitutions (Pro25, Pro28, and Pro29) in cagrilintide suppress the development of amyloid fibrils. Furthermore, the Tyr37 Pro substitution boosts efficacy. The peptide also contains two substitutions: Asn14 Glu, which prevents deamination, and Val17 Arg, which increases solubility at physiological pH and forms a helix-stabilizing salt bridge with Glu14. Additionally, the attachment of a C-20 fatty diacid via α-glutamyl spacer increases the duration of action by binding to albumin [82]. A summary of the available studies of cagrilintide treatment in patients with diabesity is presented in Table 2.

A recent phase 2 trial by Frias et al. showed for the first time the effect of co-administered cagrilintide and semaglutide (GLP-1 receptor agonist) on glycemic control in T2DM patients with a body mass index of 27 kg/m^2^ or higher who were treated with metformin with or without an SGLT2 inhibitor. Treatment with the combination therapy, CagriSema, resulted in clinically significant improvements in glycemic control. The mean changes in HbA_1c_ and TIR were greater compared to cagrilintide but not semaglutide. However, the use of CagriSema resulted in significantly greater weight loss compared to both semaglutide and cagrilintide [83].

Although the trial mentioned above is the only completed study of cagrilintide therapy in T2DM, another study by Lau et al. investigated its use in overweight and obese patients without T2DM. Obese or overweight patients were given a weekly subcutaneous injection of cagrilintide (0.3–4.5 mg/week), which reduced body weight by 6.4–11.5 kg compared to patients in a placebo group (3.3 kg weight loss). In the same study, the effect of cagrilintide was compared with a placebo and liraglutide. The mean percentage weight reductions were greater in all doses of cagrilintide (6.0–10.8%) in contrast to placebo (3.0%). Moreover, weight loss was also more prominent in the cagrilintide (10.8%) group compared to the liraglutide group (9.0%) [88]. Similarly, another study by Enebo et al. demonstrated weight loss (15.4%) after 20 weeks of cagrilintide therapy (4.5 mg/week) in combination with semaglutide (2.4 mg/week) [89].

## 4. Conclusions

Overall, the current evidence suggests that both pramlintide and cagrilintide are safe, effective, and promising drugs that successfully reduce body weight in patients with T2DM and consequently regulate glucose homeostasis. We expect that the results of many critical ongoing studies will shed further light on the clinical pharmacology and therapeutic potentials of amylin and its analogs. One of the potentially beneficial therapeutic possibilities is the combination of cagrilintide and semaglutide, CagriSema, which is being tested in several studies with diabesity patients.

## Figures and Tables

**Table 2:** Summary of the studies in patients with diabesity treated with cagrilintide. T1DM—type 1 diabetes mellitus; T2DM—type 2 diabetes mellitus; HbA1C—hemoglobin A1C.

Authors	Research Design (Duration, Comparator)	Number of Patients	Results	Main Findings
Frias J P et al., 2023 [83]	Multicenter, double-blinded randomized study. The safety and effect of cagrilintide and semaglutide combination in **T2DM** patients. Duration: one year.	Patients were divided into three groups: 31 with CagriSema, 31 with semaglutide, and 30 with cagrilintide	CagriSema had a significant HbA1c reduction compared with cagrilintide (−1.3%) but not with semaglutide (−0.4%). CagriSema had better results regarding the change in body weight (−15.6%) in comparison with cagrilintide –(8.1%) and semaglutide (−5.1%).	CagriSema therapy led to better glycaemic control in T2DM patients. CagriSema had better results in HBA1c reduction than cagrilinitide but not semaglutide. CagriSema had the best results regarding weight loss.
NCT06065540 Study start: 2023 [84]	The study is randomized with parallel assignment. The effect of CagriSema, cagrilintide, semaglutide, and placebo on blood glucose and body weight) in **T2DM** patients treated with metformin.	Estimated enrollment of 2700 participants	Not available	Ongoing study
NCT05394519 Study start: 2023 [85]	The study is randomized with parallel assignment. The effect of CagriSema on weight loss in patients with excess body weight and **T2DM**.	Estimated enrollment of 1200 participants	Not available	Ongoing study
NCT06131372 Study start: 2024 [86]	Randomized with parallel assignment. Comparison between CagriSema, cagrilintide, semaglutide, and placebo regarding the renal damage in people with chronic kidney disease, **T2DM**, and **excess body weight**.	Estimated enrollment of 618 participants	Not available	Ongoing study
NCT05567796 Study start: 2022 [87]	The study is randomized with parallel assignment. Safety and effectiveness of CagriSema in patients with **excess body weight**.	Estimated enrollment of 3400 participants	Not available	Ongoing study

## Data Availability

Not applicable.
